# COVID-19 vaccine acceptance and related behavioral and psychological characteristics in individuals with mental disorders in Korea

**DOI:** 10.3389/fpsyt.2023.1195103

**Published:** 2023-05-16

**Authors:** Seunghyong Ryu, Hangoeunbi Kang, Ha-Ran Jung, Hyunju Yun, Shi-Hyun Kang, Tae-Suk Kim, Seunggi Choi, Ju-Wan Kim, Ju-Yeon Lee, Jae-Min Kim, Sook-In Jung, Bo-Hyun Yoon, Sung-Wan Kim

**Affiliations:** ^1^Department of Psychiatry, Chonnam National University Medical School, Gwangju, Republic of Korea; ^2^Department of Psychiatry, Naju National Hospital, Naju, Republic of Korea; ^3^Gokseonggun Mental Health Center, Gokseong, Republic of Korea; ^4^Department of Psychiatry, Seoul National Hospital, Seoul, Republic of Korea; ^5^Department of Psychiatry, Seoul St. Mary's Hospital, The Catholic University of Korea College of Medicine, Seoul, Republic of Korea; ^6^Mindlink, Gwangju Bukgu Mental Health Center, Gwangju, Republic of Korea; ^7^Department of Infectious Diseases, Chonnam National University Medical School, Gwangju, Republic of Korea

**Keywords:** COVID-19, mental disorders, COVID-19 vaccines, vaccine hesitancy, cluster analysis

## Abstract

**Objective:**

This study aimed to investigate COVID-19 vaccine acceptance and related factors in individuals with mental disorders in Korea.

**Methods:**

We surveyed 572 individuals with mental disorders about their attitudes toward COVID-19 vaccination using a 7-item self-rating questionnaire on vaccine acceptance and hesitancy. We categorized the respondents into groups based on their level of vaccine acceptance using hierarchical clustering. In addition, we evaluated the respondents’ vaccination status and trust in sources of information regarding COVID-19 vaccines, and assessed their psychological characteristics using the Patient Health Questionnaire-9, Gratitude Questionnaire-6, and Big Five Inventory-10.

**Results:**

Clustering revealed three groups according to vaccine acceptance: ‘totally accepting’ (*n*= 246, 43.0%), ‘somewhat accepting’ (*n*= 184, 32.2%), and ‘hesitant’ (*n*= 142, 24.8%) groups. Three quarters of all participants, who belonged to the ‘totally accepting’ or ‘somewhat accepting’ groups, were willing to receive a COVID-19 vaccine despite concerns about its side effects. Individuals in the high vaccine acceptance group were older (*F*= 12.52, *p*< 0.001), more likely to receive the influenza vaccine regularly, and more likely to trust formal information sources. Additionally, they had higher levels of gratitude (*F*= 21.00, *p*< 0.001) and agreeableness (*F*= 4.50, *p*= 0.011), and lower levels of depression (*χ*^2^= 11.81, *p*= 0.003) and neuroticism (*F*= 3.71, *p*= 0.025).

**Conclusion:**

The present study demonstrated that individuals with mental disorders were generally willing to receive COVID-19 vaccination. However, they weighed its need and effectiveness against potential side effects before coming to a decision. It is important to understand the behavioral and psychological characteristics associated with vaccine acceptance, to effectively communicate its importance to individuals with mental disorders.

## Introduction

Coronavirus disease 2019 (COVID-19) emerged as a global health issue in early 2020. Compulsory public health measures, including mandatory face mask wearing and social distancing, were implemented during the early period of the pandemic to curtail the rapid spread of COVID-19 (1, 2). Almost a year after the pandemic began, COVID-19 vaccines showing promising efficacy and safety were developed, and government authorities strongly encouraged as many people as possible to be vaccinated (3). Such stringent measures were necessary to reduce morbidity and mortality among older individuals and patients with medical comorbidities. However, some individuals felt that their personal freedoms were violated and raised concerns about the efficacy and safety of the vaccines (4).

Preexisting mental disorders have been associated with a disproportionately higher likelihood of contracting COVID-19, and being hospitalized or dying, compared to the general population (5). Several factors might contribute to the poor COVID-19 outcomes of individuals with mental disorders, including a higher prevalence of physical comorbidities, unhealthy lifestyle, and immunological disturbances related to the psychopharmacological treatments (6, 7). Many individuals with mental disorders also have adverse socioeconomic conditions, which make it difficult to access appropriate physical healthcare (8). In particular, patients in closed psychiatric wards are likely to have an increased risk of contracting COVID-19 due to the overcrowded and closed nature of the environment (9, 10). In this regard, individuals with mental disorders have been considered one of the most vulnerable populations to COVID-19, and in urgent need of COVID-19 vaccination (11, 12). A longitudinal cohort study found that COVID-19 vaccination can significantly reduce COVID-19-related hospitalization and mortality rates in patients with schizophrenia to levels comparable to the general population (13).

Despite the urgent need for COVID-19 vaccination, individuals with mental disorders may be reluctant to receive the vaccine due to socioeconomic inequalities, including lower income and education levels, impaired function, and social isolation (14, 15). Psychological conditions may also significantly influence their perceptions about COVID-19 vaccination (16). However, there are limited studies on the willingness, hesitancy or reluctance of individuals with mental disorders to get vaccinated, and the extent of vaccine acceptance in this population is not well understood (17, 18). Since vaccine acceptance is a complex outcome behavior resulting from a decision-making process, it is necessary to comprehensively investigate the attitudes and behaviors of individuals with mental disorders toward COVID-19 vaccination. (19, 20).

In Korea, COVID-19 vaccination was initiated at the end of February 2021, with priority given to individuals with mental disorders (21). This study aimed to investigate COVID-19 vaccine acceptance and related factors in this population. We first examined attitudes toward vaccination and then used clustering analysis to identify patterns of vaccine acceptance. We also explored behavioral and psychological characteristics associated with vaccine acceptance. The results provided a detailed understanding of COVID-19 vaccine acceptance in individuals with mental disorders, including vaccine acceptance rates, vaccination behaviors, and related psychological factors.

## Materials and methods

### Participants

This study enrolled 663 individuals with mental disorders from two university hospitals (277 outpatients), two mental hospitals (206 outpatients), and two community mental health centers (180 individuals) in South Korea between August 2 and December 31, 2021. Participants were aged 19–70 years, presented to the psychiatric outpatient clinic or community mental health center, were able to provide informed consent and complete the questionnaire. The potential participants were selected using non-probability sampling. A psychiatrist, psychologist, or mental health social worker explained the study procedures to the participants and obtained written informed consent prior to the completion of self-rated questionnaires. In total, 572 participants were included in the analysis, after excluding 91 who did not complete the questionnaires or had missing demographic data. The study was approved by the Chonnam National University Hospital Institutional Review Board (CNUH-2021-297).

### Measures

The participants indicated their acceptance and hesitancy with regard to COVID-19 vaccination via seven items on a COVID-19 vaccination attitude questionnaire that we developed based on existing literature and our experience. The responses were rated using a 5-point Likert scale ranging from 1 (*Strongly disagree*) to 5 (*Strongly agree*; Table 1). The internal consistency of this questionnaire was acceptable (Cronbach’s α = 0.75, when questions 1–3 were reverse-scored). The participants were also asked about their vaccination status for COVID-19 and influenza vaccines, and then only those who had already received the COVID-19 vaccine or were scheduled to receive it shortly, responded to six Yes or No questions regarding their reasons for receiving the vaccine. In addition, all participants were asked six Yes or No questions about trustworthy sources of information regarding COVID-19 vaccination.

**Table 1:** COVID-19 vaccination attitude questionnaire.

No	Item contents
1	I am worried that the COVID-19 vaccination will cause side effects.
2	I am afraid of getting an injection.
3	I do not need COVID-19 vaccination.
4	I am willing to receive COVID-19 vaccination annually, if necessary.
5	I think that the benefit of COVID-19 vaccination outweighs the risks of side effects.
6	I am willing to recommend COVID-19 vaccination to individuals around me.
7	I think COVID-19 vaccines effectively prevent COVID-19.

All items on the questionnaires were rated using a 5-point Likert scale (1, strongly disagree; 2, disagree; 3, neutral; 4, agree; 5, strongly agree).

Depression was measured using the Patient Health Questionnaire (PHQ)-9 (22). The PHQ-9 items were scored based on frequency using a 4-point Likert scale ranging from 0 (*not at all*) to 3 (*nearly every day*). A cutoff score of ≥10 indicates clinically relevant symptoms of depression. We used the Korean version of the PHQ-9, which is a reliable and valid tool for screening depressive symptoms in Korean populations (23). In this study, the Cronbach’s α of the PHQ-9 was 0.91, indicating acceptable internal consistency.

Gratitude was assessed using the Gratitude Questionnaire (GQ)-6, which evaluates the experience and expression of gratitude in daily life (24). The GQ-6 items were rated on a 7-point Likert scale ranging from 1 (*strongly disagree*) to 7 (*strongly agree*). Higher scores indicate more grateful attitudes and more positive emotions. We used the Korean version of the GQ-6, which has demonstrated high reliability and validity (25). In this study, the Cronbach’s α of the GQ-6 was 0.87, indicating acceptable internal consistency.

Personality traits were assessed using the Big Five Inventory (BFI)-10, which is a short-form version of the BFI that measures five dimensions of personality, including extraversion, agreeableness, conscientiousness, neuroticism, and openness to experience (26). The BFI-10 items were rated on a 5-point Likert scale ranging from 1 (*disagree strongly*) to 5 (*agree strongly*). The score for each personality dimension was calculated as the sum of the normal score question and reverse score question. We used the Korean version of the BFI-10, which has been validated with good reliability and validity (27).

### Statistical analysis

We performed cluster analysis on the responses to the seven questions comprising the COVID-19 vaccination attitude questionnaire, to identify a set of individuals with similar levels of vaccine acceptance. The 5-point Likert scale scores were treated as ordinal variables and subjected to hierarchical clustering using Ward’s minimum variance method, which minimizes the total variance within each cluster. Gower’s distance was used as a dissimilarity matrix, suitable for ordinal variables (28). Hierarchical clustering constructs a dendrogram of nested clusters by repeatedly merging or splitting clusters (29). We determined the optimal number of clusters using both the elbow and silhouette methods. The elbow method considers only intra-cluster distances, while the silhouette method uses a combination of inter-and intra-cluster distances, which may lead to different results (30). We visualized individual response patterns to determine properties of the clusters. This process was performed using the R packages ‘cluster’ and ‘factoextra’. Then, we compared vaccination behaviors and psychological characteristics among clusters, using the Chi-squared test for categorical variables and Quade non-parametric covariance analysis for covariate-adjusted continuous variables (31). All statistical tests were two-tailed. *p* < 0.05 was considered statistically significant. Statistical analyses were performed using R (version 4.0.3; R Foundation for Statistical Computing, Vienna, Austria) and SPSS software (version 27.0; IBM Corp, Armonk, NY, United States).

## Results

### Identification of clusters

**Figure 1:**
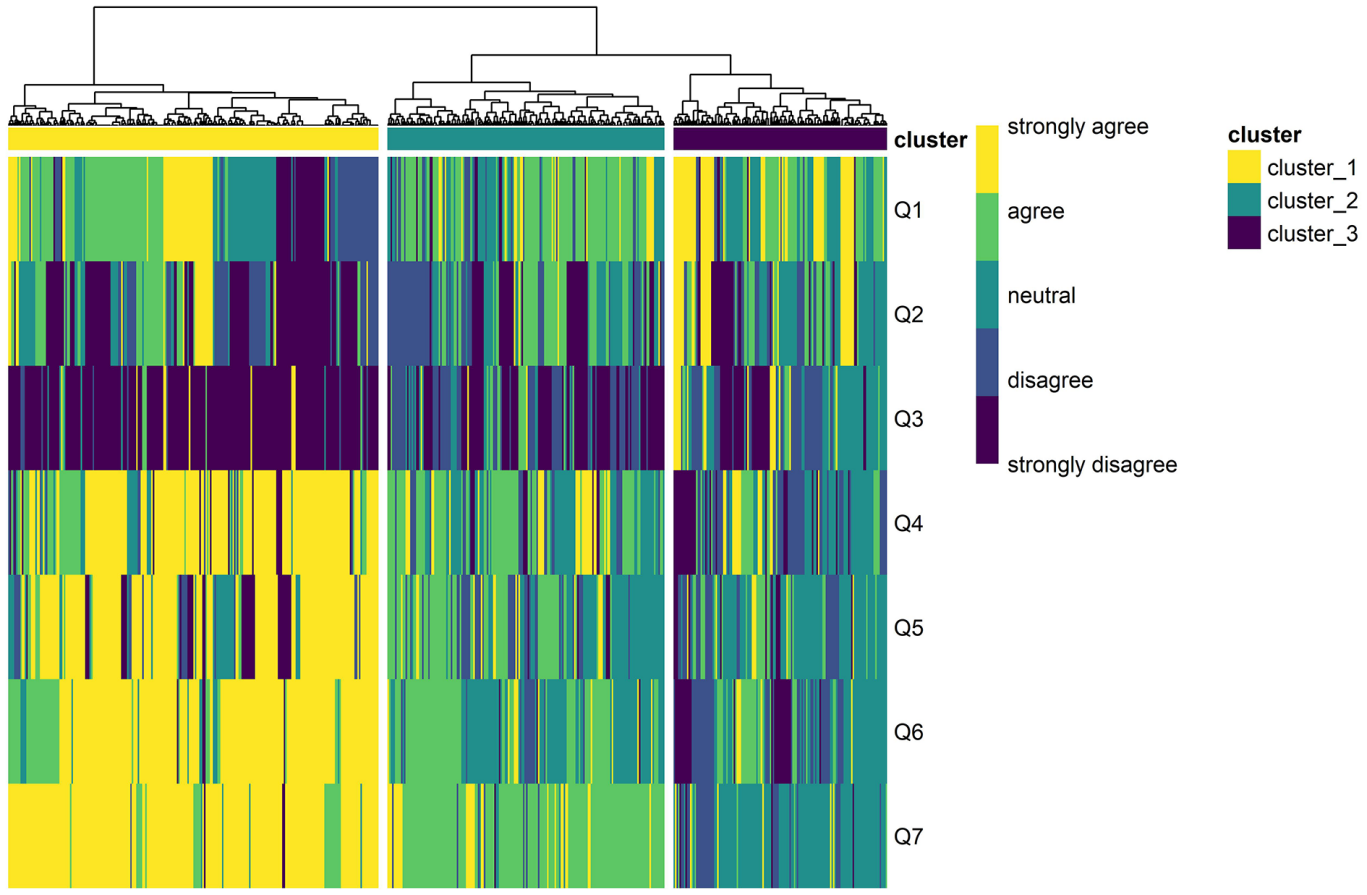
Heatmap and dendrogram of the hierarchical clustering analysis results; clusters 1–3 were distinguished based on the responses to the seven questions on attitudes toward COVID-19 vaccination of individuals with mental disorders.

The elbow and silhouette method suggested that two or three clusters would be optimal. Considering the dendrogram and heatmap, we decided to classify the study population into three clusters (Figure 1). In cluster 1 (*n* = 246, 43.0%), most respondents strongly agreed with the items related to a positive attitude toward COVID-19 vaccination (questions 4–7) and strongly disagreed with question 3 (i.e., “I do not need the COVID-19 vaccination”). Approximately half of the respondents in cluster 1 strongly agreed or agreed that they were concerned about potential side effects of the COVID-19 vaccines in question 1. Similarly, in cluster 2 (*n* = 184, 32.2%), most participants agreed with questions 4–7 and disagreed or strongly disagreed with question 3. By contrast, in cluster 3 (n = 142, 24.8%), most participants were neutral toward, or disagreed or strongly disagreed, with questions 4–7, and many of them strongly agreed or agreed with question 3. In addition, > 60% of the respondents in cluster 3 expressed concerns about side effects in question 1. Based on these patterns of responses, clusters 1–3 were labeled ‘totally accepting’, ‘somewhat accepting’, and ‘hesitant’ groups, respectively.

### Demographic characteristics

The study participants had a mean age of 36.6 ± 12.0 years, and 47% were males. The most common diagnosis was schizophrenia (58.0%), followed by depressive disorder (19.6%), bipolar disorder (11.5%), anxiety disorder (3.8%), and others (7.0%).

There were no significant differences in demographic characteristics among the three groups, except in age (Table 2). Participants in the ‘totally accepting’ group were significantly older than those in the ‘somewhat accepting’ and ‘hesitant’ groups (*F* = 12.52, *p* < 0.001). Therefore, we controlled for age as a covariate when comparing other continuous variables among the three groups.

**Table 2:** Comparison of demographic characteristics among the three COVID-19 vaccine acceptance groups.

Totally accepting	Somewhat accepting	Hesitant	Statistics^a^
Sex (male / female)			
114 / 132 (46.3 / 53.7)	91 / 93 (49.5 / 50.5)	64 / 78 (45.1 / 54.9)	*χ*^2^ = 0.70, *p* = 0.704
Age, years			
39.38 ± 12.30 (19–70)	34.47 ± 11.26 (19–69)	34.47 ± 11.26 (19–69)	*F* = 12.52, *p* < 0.001
Marital status (single / married)			
186 / 57 (76.5 / 23.5)	132 / 50 (72.5 / 27.5)	109 / 33 (76.8 / 23.2)	*χ*^2^ = 1.12, *p* = 0.572
Education (≤ 12 / > 12 years)			
95 / 150 (38.8 / 61.2)	78 / 105 (42.6 / 57.4)	58 / 84 (40.8 / 59.2)	*χ*^2^ = 0.65, *p* = 0.722
Occupation (employed / unemployed)			
86 / 157 (35.4 / 64.6)	59 / 124 (32.2 / 67.8)	48 / 91 (34.5 / 65.5)	*χ*^2^ = 0.47, *p* = 0.790
Medical insurance (health insurance / Medicare)			
169 / 68 (71.3 / 28.7)	140 / 39 (78.2 / 21.8)	101 / 35 (74.3 / 25.7)	*χ*^2^ = 2.54, *p* = 0.280
Diagnosis (schizophrenia and bipolar disorder / depressive and anxiety disorder / other)			
172 / 56 / 18 (69.9 / 22.8 / 7.3)	130 / 42 / 12 (70.7 / 22.8 / 6.5)	96 / 36 / 10 (67.6 / 25.4 / 7.0)	*χ*^2^ = 0.51, *p* = 0.973

Data are presented as number (%) or mean ± standard deviation (range). Some data were missing.

a Chi-square test or ANOVA.

### Vaccination behaviors

At the time of the survey, almost 50–60% of participants in the ‘totally accepting’ and ‘somewhat accepting’ groups had already received the COVID-19 vaccine (Table 3A). However, only 33% of participants in the ‘hesitant’ group had been vaccinated. The proportion of participants who regularly received the influenza vaccine was lower in the ‘hesitant’ group (< 20%) compared to the other two groups.

**Table 3:** Comparisons of vaccination behaviors among the three COVID-19 vaccine acceptance groups.

Totally accepting	Somewhat accepting	Hesitant	Statistics^a^
**A. Vaccination status**			
COVID-19 vaccination (already vaccinated / soon to be vaccinated / not yet vaccinated)			
154 / 73 / 19 (62.6 / 29.7 / 7.7)	90 / 61 / 33 (48.9 / 33.2 / 17.9)	47 / 46 / 49 (33.1 / 32.4 / 34.5)	*χ*^2^ = 52.65, *p* < 0.001
Influenza vaccination (vaccinated every year / vaccinated every few years / seldom vaccinated)			
88 / 72 / 86 (35.8 / 29.3 / 35.0)	48 / 62 / 74 (26.1 / 33.7 / 40.2)	25 / 43 / 74 (17.6 / 30.3 / 52.1)	*χ*^2^ = 18.17, *p* = 0.001
**B. Reasons for receiving COVID-19 vaccination** (yes / no)^b^			
To prevent COVID-19 infection			
198 / 26 (88.4 / 11.6)	112 / 37 (75.2 / 24.8)	57 / 36 (61.3 / 38.7)	*χ*^2^ = 30.54, *p* < 0.001
To prevent people around me from getting infected			
148 / 76 (66.1 / 33.9)	91 / 58 (61.1 / 38.9)	44 / 49 (47.3 / 52.7)	*χ*^2^ = 9.71, *p* = 0.008
To avoid quarantine			
54 / 170 (24.1 / 75.9)	26 / 123 (17.4 / 82.6)	8 / 85 (8.6 / 91.4)	*χ*^2^ = 10.61, *p* = 0.005
To enjoy unrestricted activities of daily living			
116 / 108 (51.8 / 48.2)	72 / 76 (48.6 / 51.4)	35 / 58 (37.6 / 62.4)	*χ*^2^ = 5.32, *p* = 0.070
Recommended by those around me			
22 / 202 (9.8 / 90.2)	26 / 123 (17.4 / 82.6)	20 / 73 (21.5 / 78.5)	*χ*^2^ = 8.63, *p* = 0.013
Following those around me who got vaccinated			
60 / 164 (26.8 / 73.2)	43 / 106 (28.9 / 71.1)	25 / 68 (26.9 / 73.1)	*χ*^2^ = 0.21, *p* = 0.899
**C. Trust in information sources regarding the COVID-19 vaccination** (yes / no)			
Internet news			
103 / 141 (42.2 / 57.8)	64 / 117 (35.4 / 64.6)	46 / 95 (32.6 / 67.4)	*χ*^2^ = 4.09, *p* = 0.130
Internet videos (e.g., YouTube)			
43 / 201 (17.6 / 82.4)	26 / 155 (14.4 / 85.6)	20 / 121 (14.2 / 85.8)	*χ*^2^ = 1.17, *p* = 0.558
TV and radio news			
168 / 76 (68.9 / 31.1)	99 / 82 (54.7 / 45.3)	71 / 70 (50.4 / 49.6)	*χ*^2^ = 15.50, *p* < 0.001
Social network services			
24 / 220 (9.8 / 90.2)	22 / 159 (12.2 / 87.8)	8 / 133 (5.7 / 94.3)	*χ*^2^ = 3.90, *p* = 0.142
Acquaintances (family, friends, etc.)			
74 / 170 (30.3 / 69.7)	52 / 129 (28.7 / 71.3)	46 / 95 (32.6 / 67.4)	*χ*^2^ = 0.57, *p* = 0.752
Medical professionals			
144 / 100 (59.0 / 41.0)	96 / 85 (53.0 / 47.0)	54 / 87 (38.3 / 61.7)	*χ*^2^ = 15.50, *p* < 0.001

Data are presented as the number (%). Some data were missing.

a Chi-square test.

b Among those who had already received COVID-19 vaccination or were scheduled to be vaccinated soon.

Among participants who had already received the COVID-19 vaccine or were scheduled to receive it soon, those in the ‘totally accepting’ and ‘somewhat accepting’ groups were more likely to state that prevention of infection and exemption from quarantine or other restrictions were the major reasons for receiving a COVID-19 vaccine compared to those in the ‘hesitant’ group (Table 3B). However, the proportion of those participants who had been vaccinated against their will was significantly higher in the ‘hesitant’ compared to ‘totally accepting’ group.

More than half of the participants in the ‘totally accepting’ and ‘somewhat accepting’ groups stated that they trusted the information related to COVID-19 vaccination presented on TV and radio news, as well as by medical professionals (Table 3C). However, in the ‘hesitant’ group, the proportion of participants who trusted these sources of information was significantly lower than in the other two groups. Additionally, individuals with mental disorders were less likely to trust information provided by online videos, social network services, and acquaintances, regardless of their vaccine acceptance status.

### Psychological characteristics associated with COVID-19 vaccine acceptance

The total PHQ-9 score was not significantly different among the three groups (*F* = 2.80, *p* = 0.062), but the proportion of participants who had clinically significant depression (i.e., PHQ-9 score ≥ 10) was significantly lower in the ‘totally accepting’ group (20.7%) compared to the ‘somewhat accepting’ (33.2%) and ‘hesitant’ (34.5%) groups (*χ*^2^ = 11.81, *p* = 0.003; Table 4A).

**Table 4:** Comparison of psychological characteristics among the three COVID-19 vaccine acceptance groups.

Totally accepting	Somewhat accepting	Hesitant	Statistics^a^
**A. Depression (Patient health questionnaire-9)**			
Total score			
6.02 ± 6.56	7.02 ± 6.45	7.42 ± 6.86	*F* = 2.80, *p* = 0.062
Score of <10 / ≥10			
195 / 51 (79.3 / 20.7)	123 / 61 (66.8 / 33.2)	93 / 49 (65.5 / 34.5)	χ^2^ = 11.81, *p* = 0.003
**B. Gratitude (Gratitude questionnaire-6)**			
32.98 ± 7.54	29.30 ± 7.20	27.96 ± 7.96	*F* = 21.00, *p* < 0.001^b^
**C. Personality traits (Big five inventory-10)**			
Extraversion			
5.90 ± 1.70	5.89 ± 1.51	5.75 ± 1.53	*F* = 0.28, *p* = 0.753
Agreeableness			
7.13 ± 1.54	6.82 ± 1.27	6.62 ± 1.29	*F* = 4.50, *p* = 0.011^c^
Conscientiousness			
6.50 ± 1.98	6.17 ± 1.54	6.13 ± 1.44	*F* = 0.74, *p* = 0.476
Neuroticism			
5.69 ± 1.98	5.81 ± 1.50	6.19 ± 1.64	*F* = 3.71, *p* = 0.025^d^
Openness to experience			
7.07 ± 1.86	6.85 ± 1.68	6.82 ± 1.76	*F* = 2.86, *p* = 0.058

Data are presented as mean ± standard deviation or number (%).

a Quade’s nonparametric ANCOVA (including age as a covariate) or chi-square test.

b Totally accepting > Somewhat accepting, Totally accepting > Hesitant.

c Totally accepting > Hesitant.

d Totally accepting < Hesitant, Somewhat accepting < Hesitant.

The total GQ-6 score was significantly different among the three groups (*F* = 21.00, *p* < 0.001; Table 4B). *Post hoc* tests showed that the ‘totally accepting’ group had a higher level of gratitude compared to the ‘somewhat accepting’ and ‘hesitant’ groups.

Regarding the BFI-10 scores, there were significant differences in agreeableness (*F* = 4.50, *p* = 0.011) and neuroticism (*F* = 3.71, *p* = 0.025) among the three groups (Table 4C). *Post hoc* tests showed that the level of agreeableness was higher in the ‘totally accepting’ than ‘hesitant’ group, and the level of neuroticism was higher in the ‘hesitant’ than ‘totally accepting’ and ‘somewhat accepting’ groups.

## Discussion

Our clustering analysis showed that three-quarters of the participants (75.2%) accepted COVID-19 vaccination, and perceived it as efficacious and necessary. However, the remaining participants (24.8%) were reluctant to get vaccinated and were overly concerned about side effects. We also analyzed the demographic factors, motivations, trust in information sources, and psychological characteristics associated with vaccine acceptance.

### Hierarchical clustering for COVID-19 vaccine acceptance in individuals with mental disorders

This study aimed to identify COVID-19 vaccine acceptance in individuals with mental disorders by examining their concerns, needs, and motivations for vaccination. Only a few studies have been conducted on COVID-19 vaccine acceptance in this population, and these studies have often relied on one or two simple questions about vaccination intent, such as “Do you intend to be vaccinated against COVID-19 in the future?” or “Will you accept vaccination against coronavirus, once it is offered to you?” (17, 18). However, vaccine decision-making is a complex process that involves an individual’s values, background, and coping strategies (19, 20). Therefore, to gain a more comprehensive understanding of vaccine acceptance in individuals with mental disorders, we utilized a wider range of questions about COVID-19 vaccination and a clustering method instead of relying solely on simple questions and analyses based on fixed cut-off scores. Clustering is an exploratory analysis technique used to identify subgroups of individuals within a larger population who share similar characteristics (32). When validated tools to assess vaccination behaviors in a specific population are not available, clustering analysis can be used for data-driven categorization of the population according to COVID-19 vaccine acceptance or hesitancy. A recent study based on cluster analysis found that patients with autoimmune and inflammatory diseases were characterized by three main patterns of beliefs and intentions related to COVID-19 vaccination (33).

In the present study, hierarchical clustering identified three main types of attitudes toward COVID-19 vaccination in individuals with mental disorders: ‘totally accepting’, ‘somewhat accepting’, and ‘hesitant’. Most participants in the ‘totally accepting’ group strongly agreed that COVID-19 vaccines are efficacious and necessary, and expressed high willingness to be vaccinated. Additionally, many participants in the ‘somewhat accepting’ group were somewhat willing to be vaccinated, and agreed with the necessity of vaccination. However, a considerable proportion of participants in both the ‘totally accepting’ and ‘somewhat accepting’ groups expressed concerns about potential side effects. The ‘totally accepting’ and ‘somewhat accepting’ groups accounted for three-quarters of all participants (75.2%). This vaccine acceptance rate was lower than that reported in a Danish study of mental disorder patients (84.8%) (18), but was higher than that reported in a Chinese study (50.8%) (17). These discrepancies may be because of differences in the measure of vaccine acceptance, survey timing, and study populations. By contrast, most participants in the ‘hesitant’ group (24.8%) were neutral regarding the prospect of receiving the COVID-19 vaccine, or were reluctant to receive it, and were also highly concerned about side effects.

Altogether, our clustering analysis showed that vaccine acceptance was influenced by the perceived necessity of the vaccine and concerns about potential side effects. We found that the majority of individuals with mental disorders in Korea were willing to receive the COVID-19 vaccines despite concerns about side effects. However, some individuals expressed doubts about the necessity and efficacy of the vaccines.

### Demographic characteristics associated with COVID-19 vaccine acceptance

Participants in the ‘totally accepting’ group were older than those in the ‘somewhat accepting’ and ‘hesitant’ groups. Our results are largely consistent with those of recent studies showing that older people were more willing to get vaccinated (34, 35). The higher vaccine acceptance among older patients may be due to their awareness of worse COVID-19 outcomes in the unvaccinated or higher prevalence of comorbid physical illness (36). In contrast, young people who are generally healthy and have been less affected by COVID-19 may be less inclined to receive the vaccines (37). On the other hand, we found no differences between the three groups in terms of sex, level of education, occupational status, and diagnosis. Recent studies of the general population have shown mixed results regarding the effects of these factors on vaccine acceptance. Some studies have shown that men and employed individuals are more likely to accept COVID-19 vaccines compared to women and unemployed individuals (38, 39), while others have reported the opposite (34, 40). A global survey reported that vaccine hesitancy was associated with a lower education level, while vaccine refusal was associated with a higher education level (14). The effects of mental disorder diagnosis and severity on vaccination behaviors also remain unclear. Further investigation is needed to understand the differences in vaccine acceptance based on demographic and clinical characteristics.

### Vaccination behaviors associated with COVID-19 vaccine acceptance

Our ‘hesitant’ group had the lowest rate of past influenza vaccination as well as current COVID-19 vaccination, suggesting that existing perceptions and attitudes toward vaccination might play important roles in the decision to receive a COVID-19 vaccine. COVID-19 vaccine hesitancy in the general population has been associated with not obtaining an influenza vaccination (41, 42). A systematic review also pointed out concerns over safety, lack of trust, lack of need for vaccination, and cultural reasons as common causes of vaccine hesitancy for COVID-19 and influenza vaccines (43). We speculate that negative perceptions of vaccines may underlie the hesitation or reluctance to receive COVID-19 or influenza vaccines.

Among participants who were accepting of COVID-19 vaccination, prevention of COVID-19 infection and exemption from restrictions on daily life were important factors in the decision to be vaccinated. This shows that, in addition to the prevention of infections and reduction of mortality, the benefits of vaccination for daily life might be important in the decision to be vaccinated (44). In this regard, encouraging positive perceptions and attitudes toward COVID-19 vaccines in individuals with mental disorders may increase the likelihood of COVID-19 vaccination.

Participants who exhibited high vaccine acceptance considered traditional mass media and medical professionals as reliable information sources. However, participants who were hesitant to receive the COVID-19 vaccine had less trust in these information sources. A recent study from Singapore found that trust in formal rather than informal sources of information was associated with complete vaccination among middle-aged and older individuals (45). A Swiss study found that institutional trust plays a strong role in the decision to be vaccinated (46). It is not clear whether individuals with greater vaccine acceptance are more likely to trust formal sources of information or vice versa. Additionally, vaccine acceptance in individuals with mental disorders may be influenced by the types of sources they have access to (47). Those who had more access to formal information or less access to informal information may have been more willing to get vaccinated (48). Nevertheless, providing appropriate formal or informal information on COVID-19 vaccination to individuals with mental disorders is important to increase their vaccine acceptance (49, 50).

Overall, our findings suggest that public health strategies effectively communicating the necessity and benefits of COVID-19 vaccines to individuals with mental health problems are needed.

### Psychological characteristics associated with COVID-19 vaccine acceptance

Among participants who were totally accepting of COVID-19 vaccination, the proportion who had experienced clinically significant depression was significantly lower, compared to the other groups. Depressed individuals are likely to become ambivalent, have reduced adaptive coping resources, and exhibit reluctance to take preventive actions against COVID-19 (51). Conversely, vaccination might reduce the perceived risk of COVID-19 and associated psychological distress (52). Given that individuals with mental disorders may be more vulnerable to experiencing COVID-19-related depression, anxiety, and stress, it is important to consider how these psychological conditions may impact their willingness to receive the COVID-19 vaccines (53–55).

Participants who were totally accepting of vaccination exhibited higher levels of gratitude. Gratitude is a general state of thankfulness and appreciation in response to the receipt of something that is valuable and meaningful to a given individual (56). Gratitude improves adaptive coping in the face of adversity (57). In particular, it was associated with better mental health during the COVID-19 pandemic, including less anxiety and depression, as well as a higher level of subjective well-being (58, 59). Although little is known regarding the effects of gratitude on vaccination behaviors during the pandemic, we assumed that grateful individuals with mental disorders might cope better with concerns about the new COVID-19 vaccines (60).

Participants who exhibited high vaccine acceptance had higher agreeableness and less neuroticism. Agreeableness refers to an individual’s level of cooperativeness and compassion; individuals with a high level of agreeableness are more likely to be warm, caring, and supportive toward others (61). By contrast, individuals with a high level of neuroticism are characterized by anxiety, sadness, and emotional instability; individuals with a high level of neuroticism feel more depressed, impulsive, and insecure (62). Several studies conducted before and after the pandemic have shown that personality traits such as agreeableness, conscientiousness, and neuroticism may influence vaccine acceptance and hesitancy in the general population (63, 64).

Taken together, our findings suggest that the psychological state and traits of individuals with mental disorders may play an important role in the willingness to receive the COVID-19 vaccine.

## Limitations

This study had some methodological limitations. First, the present study included only community-dwelling patients, recruited through non-probability sampling, who may not be representative of the mental disorder population. Therefore, the results should be interpreted cautiously. Second, the study population was heterogeneous in terms of underlying psychotic and neurotic diseases. Although there was no association between the underlying diagnosis and vaccine acceptance, future studies are warranted to investigate vaccination behavior in the context of individual psychiatric disorders. Third, because the study population was grouped using a clustering method rather than based on cutoff scores or criteria, the clusters in the present study did not fully reflect the absolute level of vaccine acceptance. In addition, the study did not differentiate between vaccine hesitancy and vaccine refusal. Data-driven approaches allow classification of vaccine acceptance based on the study population and investigator judgment. Fourth, the associations between vaccine acceptance and behavioral and psychological characteristics do not indicate causation direction. Further studies are needed to examine causal relationships. Fifth, this study was conducted over several months during the COVID-19 vaccination program. Therefore, temporal changes in the vaccination rates and the phasic nature of the program should be taken into consideration. Longitudinal studies are needed to understand the changes in attitudes and behavior toward COVID-19 vaccination before and after the vaccination program.

## Conclusion

In this study, we found that the majority of individuals with mental disorders were willing to receive the newly developed COVID-19 vaccines. However, some remained doubtful about the need for vaccination and were overly concerned about vaccine side effects. Perceptions of the efficacy and necessity of COVID-19 vaccines varied among this population. Additionally, the way individuals weighed the benefits and risks of vaccination may have influenced their acceptance or hesitancy toward receiving the COVID-19 vaccines. Depression, gratitude, and personality characteristics also play important roles in attitudes and decisions regarding COVID-19 vaccination in individuals with mental disorders. Effective communication of objective information about COVID-19 vaccination to this population is crucial to help them understand the importance of vaccination and alleviate their concerns about potential side effects. Public health strategies should consider the behavioral and psychological characteristics of this population to improve their adherence to vaccination and reduce vaccine hesitancy or refusal.

## Data availability statement

The raw data supporting the conclusions of this article will be made available by the authors, without undue reservation.

## Ethics statement

The studies involving human participants were reviewed and approved by the Chonnam National University Hospital Institutional Review Board. The patients/participants provided their written informed consent to participate in this study.

## Author contributions

S-WK and SR have contributed to the conception, design of the study, and drafted the manuscript. HK, H-RJ, HY, S-HK, T-SK, and SC conducted the data collection. S-WK, SR, and J-WK were involved in the analysis. J-YL, J-MK, S-IJ, and B-HY critically revised the draft. All authors contributed to the article and approved the submitted version.

## Funding

This research was supported by grants of Patient-Centered Clinical Research Coordinating Center (PACEN) funded by the Ministry of Health and Welfare, Republic of Korea (grant numbers: HI19C0481 and HC19C0316).

## Conflict of interest

The authors declare that the research was conducted in the absence of any commercial or financial relationships that could be construed as a potential conflict of interest.

## Publisher’s note

All claims expressed in this article are solely those of the authors and do not necessarily represent those of their affiliated organizations, or those of the publisher, the editors and the reviewers. Any product that may be evaluated in this article, or claim that may be made by its manufacturer, is not guaranteed or endorsed by the publisher.
